# Thermal distribution, physiological effects and toxicities of extracorporeally induced whole‐body hyperthermia in a pig model

**DOI:** 10.14814/phy2.14366

**Published:** 2020-02-25

**Authors:** Gerben Lassche, Tim Frenzel, Marcel H. Mignot, Marianne A. Jonker, Johannes G. van der Hoeven, Carla M. L. van Herpen, Gert Jan Scheffer

**Affiliations:** ^1^ Department of Medical Oncology Radboud University Medical Center Nijmegen The Netherlands; ^2^ Department of Intensive Care medicine Radboud University Medical Center Nijmegen The Netherlands; ^3^ Vithèr Hyperthermia B.V. Laren The Netherlands; ^4^ Department of Health Evidence Radboud University Medical Center Nijmegen The Netherlands; ^5^ Department of Anesthesiology Radboud University Medical Center Nijmegen The Netherlands

**Keywords:** extracorporeal circulation, hemodynamics, induced hyperthermia, safety, whole‐body hyperthermia

## Abstract

**Background:**

Extracorporeally induced whole‐body hyperthermia (eWBH) might be a beneficial treatment in cancer patients. Objectives of this pig study were to assess thermal distribution, (patho‐)physiological effects, and safety of eWBH with a new WBH device.

**Methods:**

Fourteen healthy adult pigs were anesthetized, mechanically ventilated, and cannulated; 12 were included in the analysis. Blood was heated in 11 pigs (one pig served as control) using a WBH device (Vithèr Hyperthermia B.V.) containing two separate fluidic circuits and a heat exchanger. Temperature was monitored on nine different sites, including the brain. Core temperature (average of 4 deep probes) was elevated to 42°C for 2 hr.

**Results:**

Elevation of core body temperature to 42°C took on average (± standard deviation) 38 ± 8 min. Initially observed temperature spikes diminished after lowering maximal blood temperature to 45°C. Hereafter, brain temperature spikes never exceeded 42.5°C, mean brain temperature was at highest 41.9°C during maintenance. WBH resulted in increased heart rates and decreased mean arterial pressures. The vast amounts of fluids required to counter hypotension tended to be smaller after corticosteroid administration. Hemodialysis was started in three animals (potassium increase prevention in two and hyperkalemia treatment in one). Severe rhabdomyolysis was observed in all pigs (including the control). All animals survived the procedure until planned euthanasia 1, 6, or 24 hr post procedure.

**Conclusion:**

Fast induction of eWBH with homogenous thermal distribution is feasible in pigs using the Vithèr WBH device. Severe hemodynamic disturbances, rhabdomyolysis, and hyperkalemia were observed.

## Graphical abstract

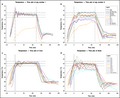

## 1 INTRODUCTION

Synergism between the beneficial effects of local or regional hyperthermia combined with chemotherapy and radiotherapy has been proven in numerous clinical studies (Datta et al., 2015; Issels, 2008; Peeken, Vaupel, & Combs, 2017) Recently, in a phase III study advantages of regional hyperthermia in combination with chemotherapy also on long‐term outcomes have been demonstrated in patients with high‐risk soft tissue sarcoma. The hazard ratio of prolonged survival rates for chemotherapy in combination with hyperthermia compared to chemotherapy alone was 0.73 (95% Confidence Interval (CI) 0.54–0.98, *p* = .04) with 10‐year overall survival of 52.6% (95% CI 44.7%–60.6%) versus 42.7% (95% CI 35.0%–50.4%) (Issels et al., 2018). Besides chemotherapy and radiotherapy sensitizing properties, hyperthermia has an intrinsic anticancer activity, of which several different pathophysiological mechanisms have been postulated. These include direct cytotoxic effects at temperatures between 41°C and 42°C, inhibition of several DNA repair mechanisms and stimulation of antitumor immunity (Oei, Vriend, Crezee, & Franken, 2015; Toraya‐Brown & Fiering, 2014; Wust et al., 2002).

Although controversial and never undisputedly confirmed, hyperthermia might be of benefit in patients with metastasized malignancies when whole‐body hyperthermia (WBH) is applied. To serve this purpose several techniques have been described to induce WBH: exogenous applied heat by submerging the body in hot fluids, radiant heating techniques, and heating the blood via extracorporeal circulation (eWBH) (Milligan, 1984; Vertrees, Leeth, Girouard, Roach, & Zwischenberger, 2002; Wust et al., 2002). The latter technique is capable of homogenous distribution of thermal energy favoring thoraco‐abdominal organs, which is essential in maintaining core body temperature within the small therapeutic window (Vertrees, Bidani, Deyo, Tao, & Zwischenberger, 2000; Vertrees et al., 2002). Consensus on the exact therapeutic window of WBH has not been reached, but is believed to be somewhere between 41°C and 43°C (Pettigrew, Galt, Ludgate, Horn, & Smith, 1974; Vertrees et al., 2002; Wust et al., 2002). It has unequivocally been proven that temperatures exceeding 43°C for more than 60 min lead to irreversible brain damage, so most of the studies on WBH aim at core body temperatures of maximal 41.8–42°C (Matsumi et al., 1994; Vertrees et al., 2002; Wust et al., 2002). Aiming at these temperatures, two studies with low number of patients proved the technique of veno‐venous inducement of eWBH to be feasible (Locker et al., 2011; Zwischenberger et al., 2004). Several manageable and reversible toxicities were observed in the first study, which included seven patients with stage IV non–small cell lung carcinoma (after initial refinement of the technique in 3 patients). These toxicities consisted of hemodynamic disturbances, atrial fibrillation, ST‐elevation, superior vena cava syndrome, confusion, somnolence, decubitus, first‐degree burn, nausea, vomiting, and pain in hip prosthesis (Zwischenberger et al., 2004). In the second study, main toxicities of 12 session of eWBH (2 hr, 41.8°C) in six patients with metastatic soft tissue sarcoma consisted of hemodynamic disturbances, elevation of liver enzymes, liver failure, thrombocytopenia, mild hemolysis, mild electrolyte disturbances, and catheter‐related problems, all reversible (Locker et al., 2011). In order to corroborate and further investigate these findings, a new device capable of inducing eWBH using a veno‐venous circulation (Vithèr WBH device) was developed, resembling earlier described techniques (Vertrees et al., 2000; Zwischenberger et al., 2004).

In this article we describe our initial experiences with this device in a pig model. Objectives were to investigate the accuracy and efficacy of thermal dose delivery, to map the thermal distribution at various organs and to assess biochemical and hemodynamic disturbances when inducing eWBH at 42°C for 2 hr with the use of the Vithèr WBH device.

## 2 MATERIALS AND METHODS

All animal experiments were conducted under active approval of the Radboud University Medical Center animal experiment committee (RU‐DEC 2011‐001). We hereby report on the experiments conducted in 12 domestic pigs. After completion of these experiments, additional experiments in two animals were conducted to corroborate compatibility of simultaneous dialysis with the WBH procedure. These two animals are not included in the analysis as data collection was incomplete for the above‐mentioned objectives.

### 2.1 Study design

Experiments were changed during the study based on the results of the prior experiments regarding observation time post procedure, maximal blood heating temperature, and the administration of corticosteroids as part of a learning curve. Table 1 summarizes the characteristics of the conducted experiments with regard to these variables. One animal received veno‐venous recirculation without WBH to serve as control (pig 9).

**Table 1:** Description of differences between experiments in different animals

Pig number	Weight (kg)	Time post procedure (hr)	Blood heating temperature (°C)	Administration of corticosteroids^a^
1	78.0	1	48	No
2	95.5	1	48	No
3	66.0	1	48	No
4	71.0	1	48	No
5	61.1	6	46	No
6	64.9	6	45	No
7	79.0	24	45	No
8	70.5	24	45	Yes
9^b^	75.0	24	—	Yes
10	72.0	24	45	Yes
11	70.0	6	45	Yes
12	69.3	6	45	Yes

a Methylprednisolone 500 mg.

b Control animal, veno‐venous extracorporeal circulation without heating.

### 2.2 Induction of anesthesia and cannulation

The procedure took place in an operating room specially equipped for animal experiments. Pigs were anesthetized using an intramuscular injection of ketamine (10 mg/kg), midazolam (1 mg/kg), and flunixin (2.2 mg/kg). Prophylactic antibiotics were administered (amoxicillin 20 mg/kg). Anesthesia was induced using propofol (2‐3 mg/kg), sufentanil (5 µg/kg), buprenorphine (10 µg/kg), atropine (50 µg/kg), and vecuronium (0.2 mg/kg). After induction pigs were endotracheally intubated and mechanically ventilated. Anesthesia was maintained using a combination of midazolam (0.6 mg kg^−1^ hr^−1^), sufentanil (10 µg kg^−1^ hr^−1^), vecuronium (0.4 mg kg^−1^ hr^−1^), and isoflurane (titrated based on mean arterial pressure). ECG electrodes were placed, as well as a pulsoximeter on the nose of the animal. End tidal carbon dioxide (etCO2) was continuously measured and ventilation parameters were adapted based on the measurements combined with arterial blood gas results. For invasive and real‐time monitoring of blood pressure, central venous pressure, cardiac output, and pulmonary artery pressure, an intra‐arterial catheter and Swan‐Ganz catheter were inserted. To create an extracorporeal circulation two large bore venous catheters were placed in the femoral vein (18 French, Medtronic EOPA 77418) and jugular vein (20 French, Medtronic EOPA 77520) via surgical cut‐down and fixed with stitches.

### 2.3 Temperature monitoring

For real‐time monitoring of temperature as feedback to the WBH device, temperature probes were placed in the bladder (Tyco 90054T), esophagus (Exacon D‐OS4A), and deep and superficially in the rectum (Exacon D‐OS4A). Core body temperature was defined as the average value of these probes. Besides this, temperature probes were placed in the right and left auditory canal (Exacon D‐TM1A), one main bronchus (Exacon D‐F1345A), the skin (Exacon D‐S18A), and the brain (Licox Ref IM3 Integra).

### 2.4 WBH device

The WBH device (Vithèr hyperthermia B.V.) contains two completely separated fluidic circuits, a blood circuit (using a centrifugal pump) and a water circuit. Both are connected to a disposable heat exchanger (Eurosets Mistral [EU5522]). The water circuit controls the heating and cooling of the extracorporeal blood to ensure blood temperature never exceeds 48°C with a maximal temperature difference between water and blood of 10°C. Blood flow (aimed at 20 ml kg min^−1^) is measured and returned blood is checked for air bubbles. The WBH device can be connected to a dialysis machine (with high flux, low flow hemofilter) to ensure rapid corrections of electrolyte disturbances if necessary.

### 2.5 WBH treatment

Heparin was used as anticoagulation, dosing guided by the Activated Clotting Time (target >200 s, measured with Medtronic ACT II). Before heating started, pigs were wrapped in an isolating blanket. As part of the earlier described learning curve blood was heated to 48°C in the first four animals and only to 45°C in the last animals. Target core body temperature was 42°C, which was maintained for 2 hr, after which active cooling to 37°C took place. Postoperative monitoring period differed between animals (range 1–24 hr, Table 1). To counter hypotension, a combination of crystalloids and colloids with norepinephrine was used to maintain adequate arterial blood pressures.

### 2.6 Euthanasia

After the postoperative surveillance period, all animals were killed using an overdose of pentobarbital according to protocol.

### 2.7 Data sampling

Temperature data of the different locations were measured and stored continuously. Vital signs (blood pressure, heart rates) were collected and blood was drawn for analysis of biochemistry, hematology, and electrolyte parameters at baseline (t = 0), at start of heating (t = 1), 15 min after start heating (t = 2), at reaching 42°C (t = 3), every 15 min in the maintenance phase (t = 4–11), 15 and 30 min after cooling started (t = 12–13), after reaching 37°C (t = 14), and hourly in the time post procedure (depending on the time post procedure t = 15 up to t = 38). Regarding hemodynamic variables normal values of pigs are comparable to human values.

### 2.8 Data analysis and statistical analysis

Regarding temperature measurements, descriptive statistics were used to express temperature data at various locations (expressing data as mean ± standard deviation [*SD*]). The time to reach goal temperature was graphically displayed using a scatterplot. The best fitting straight line based on the least squares method showed the relation between maximal blood heating temperature and time to reach goal temperature. Hemodynamic variables were individually plotted over the course to assess response of each single pig to WBH treatment, separated in groups without and with corticosteroids. Biochemical toxicities and laboratory values were expressed as median ± interquartile range (IQR) at different timepoints. No comparative statistical tests were used due to heterogeneity in treatment protocol per pig and small sample size. Statistical analysis was performed using IBM SPSS statistics software version 22.

## 3 RESULTS

Twelve pigs included in the analysis had a mean weight (±*SD*) of 72.7 ± 8.8 kg. All the 12 pigs underwent extracorporeal circulation; in 11 pigs the blood was heated (one pig served as control). All the animals survived in the experiments until euthanasia.

### 3.1 Thermal distribution

**Figure 1:**
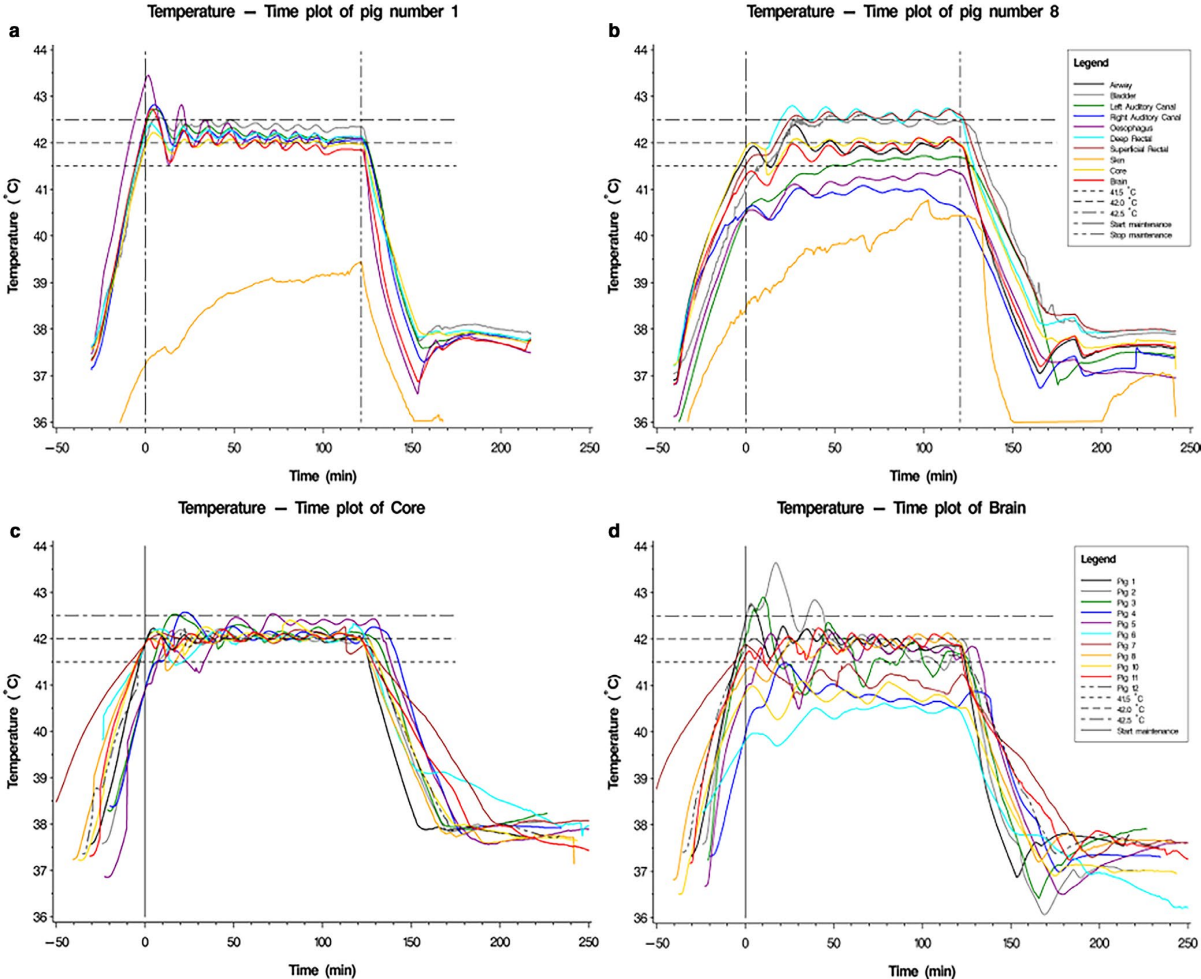
(a) Example of time–temperature curve at various locations with maximal blood heating temperature of 48°C. (b) Example of time–temperature curve at various locations with maximal blood heating temperature of 45°C. (c) Time–temperature curve of the core temperature in all pigs. (d) Time–temperature curve of the brain temperature in all pigs

**Figure 2:**
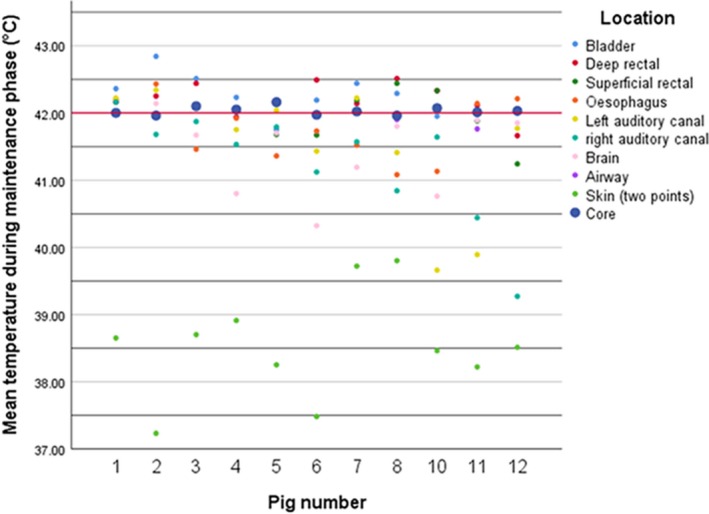
Thermal distributions at various organs during maintenance phase of 11 pigs. Red line: target temperature

**Figure 3:**
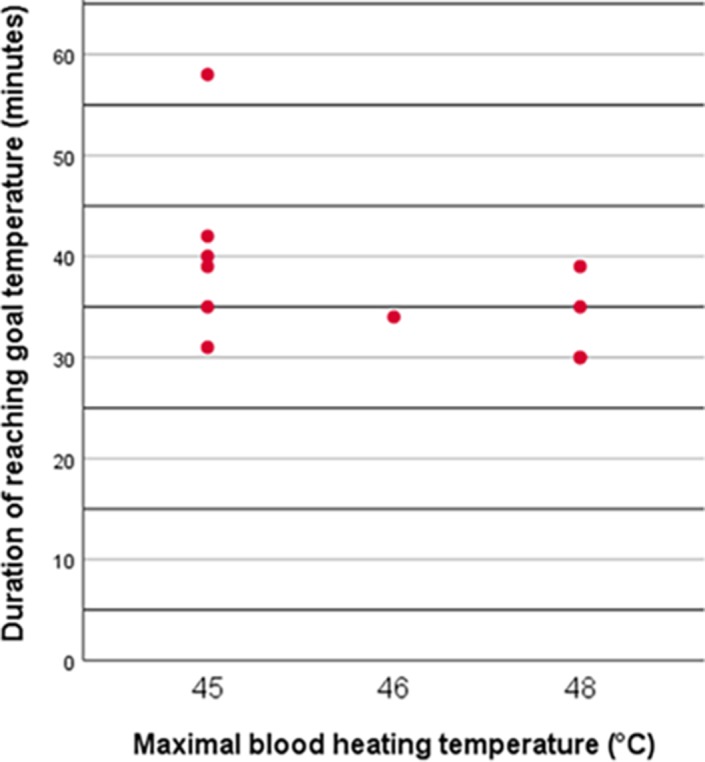
Scatterplot of individual observations of time to reach goal temperature sorted by groups with different maximal blood heating temperatures

In the first four animals blood was heated to 48°C, which led to high‐temperature peaks on several organ sites (including >43°C in the brain) shortly after reaching the core body temperature of 42°C. This observation led to lowering the maximal blood heating temperature to 45°C (initially 46°C in one animal). After this alteration temperature never exceeded 43**°**C at any of the observed locations. In Figure 1, two examples of time–temperature curves before and after adaptation of the maximal blood heating temperature are plotted (pig 1 and pig 8, Figure 1a,b); besides a time–temperature curve of the brain temperature and the core temperature in all animals (Figure 1c,d, respectively). In all the experiments with maximal blood heating temperature of 45°C, invasively monitored brain temperature during maintenance phase did not exceed 42.5**°**C and the highest mean brain temperature was 41.9 during these 2 hr at goal temperature (pig 11). Figure 2 summarizes temperature observations at various locations of the 11 heated pigs (temperature of the left auditory canal of pig 3 was not included due to improper probe placement, airway data were only available of pig 8–12). Average (±*SD*) time to reach goal temperature (42°C) was 38 ± 8 min (range 30–58 min, Figure 3). Maximal blood heating temperature only weakly correlated with the time of reaching goal temperature and could only account for 20% of the observed variation in time to reach the goal temperature. The best fitting straight line based on the least square method did not significantly better predict the time to reach the goal temperature than the mean time (*p* = .17). Individual observations of different heating times are plotted in Figure 3.

### 3.2 Hemodynamics

**Figure 4:**
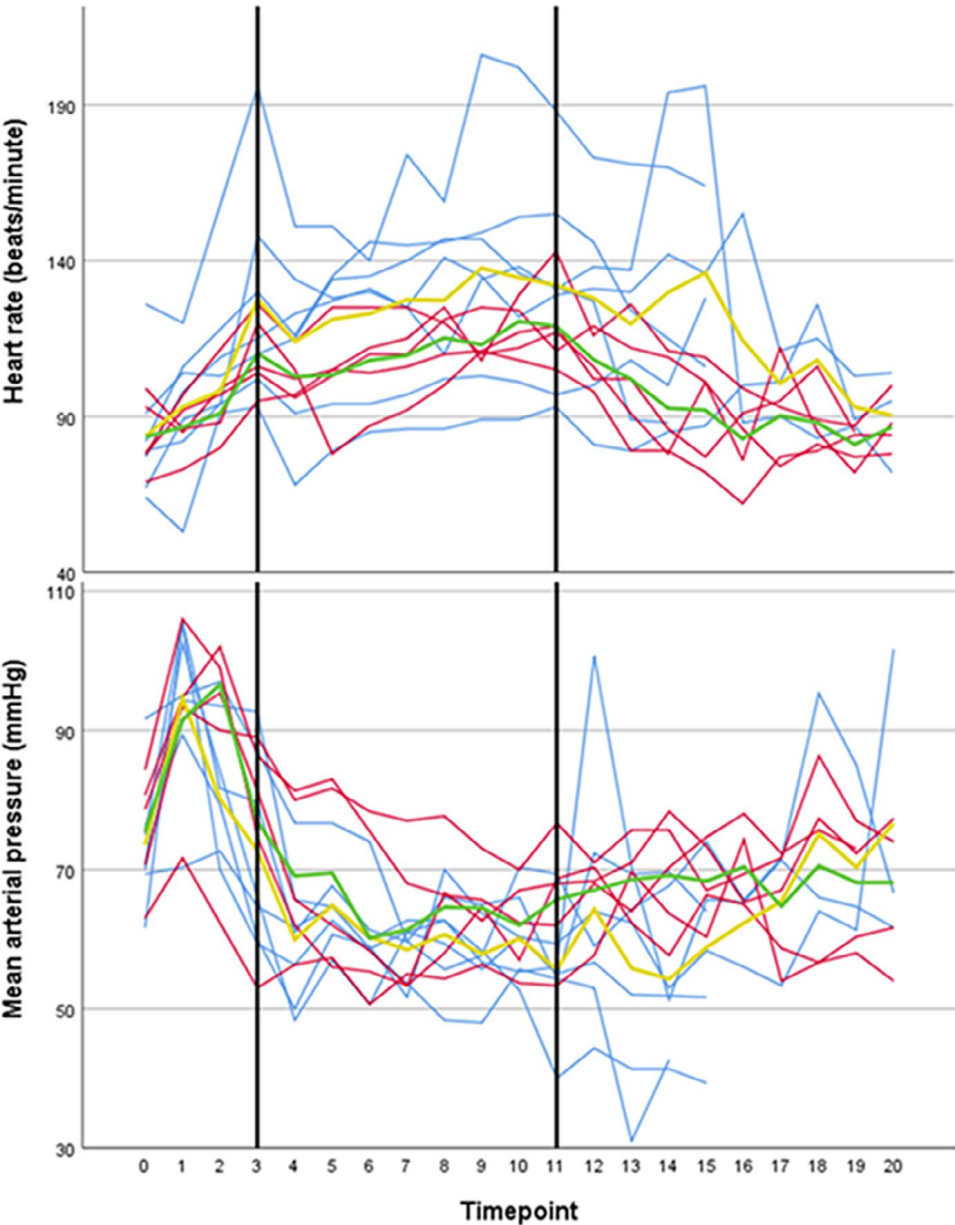
Individual courses of heart rate and mean arterial pressure until 6 hr post procedure. Black margins: maintenance phase. Blue lines: without the administration of corticosteroids, red lines: with the administration of corticosteroids, yellow line: mean without the administration of corticosteroids, green line: mean with the administration of corticosteroids. Timepoints: t = 0: baseline, t = 1: start of heating, t = 2:15 min after start heating, t = 3: reaching 42°C, t = 4–11: every 15 min in the maintenance phase, t = 12–13:15 and 30 min after cooling started, t = 14: reaching 37°C, t = 15 and higher: hourly in the time post procedure

In total, in the 12 pigs undergoing extracorporeal circulation, an increase in mean (±*SD* **)** heart rate from 84 ± 17 beats/minute (BPM) at baseline to 120 ± 25 BPM during maintenance phase was observed. On average (±*SD* **)** in all 12 pigs, arterial pressure (MAP) dropped from 74.3 ± 8.6 mmHg at baseline to 61.8 ± 8.4 mmHg during maintenance phase. Individual courses of heart rate and MAP during and after treatment are plotted for all the 12 pigs in Figure 4. However, to maintain adequate blood pressure, substantial amounts of fluid resuscitation were required. This observation, combined with the later described biochemical toxicities, led to the prolongation of the time post procedure from 1 to 6 hr initially and 24 hr eventually to investigate the recuperation of these toxicities. In total (mean ± *SD*), 6.0 ± 1.3l, 7.5 ± 1.0l, and 17.5 ± 6.5l of fluid resuscitation was administered during the procedure and the 1, 6, and 24 hr post procedure, respectively. This resulted in respective net‐positive fluid balances of 5.4 ± 1.2l, 5.4 ± 1.0l, and 8.8 ± 6.9l. Since these observations resembled the vasoplegia syndrome seen in cardiac surgery, corticosteroids (methylprednisolone 500mg) were administered in animal 8–12 in an attempt to improve vascular reactivity (Ho & Tan, 2009; Omar, Zedan, & Nugent, 2015). Table 2 summarizes the comparison between fluid balances without and with the administration of corticosteroids, showing a trend to a more stable and less positive fluid balance in the experiments with the administration of corticosteroids. As graphically displayed in Figure 4, animals receiving corticosteroids tended to perform better regarding hemodynamics, showing less fluctuations of heart rate and MAP.

**Table 2:** Comparison between fluid balances (mean ± *SD* **)** without and with corticosteroids

Time post procedure (hr)	Without corticosteroids (*N* = 7)			With corticosteroids (*N* = 5)		
	Total infused fluids (l)	Total urine production (l)	Net fluid balance (l)	Total infused fluids (l)	Total urine production (l)	Net fluid balance (l)
1 (*n* = 4/0)^a^^,^ b	6.0 ± 1.3	0.9 ± 0.6	5.4 ± 1.2	n.a.	n.a.	n.a.
6 (*n* = 2/2)^b^	7.3 ± 1.1	1.2 ± 0.5	6.0 ± 0.5	7.8 ± 1.1	3.1 ± 0.1	4.7 ± 1.1
24 (*n* = 1/3)^b^	25.5	7.1	18.4	14.8 ± 4.5	9.2 ± 2.9	5.6 ± 3.1

a Data concerning infused fluid in pig 1 were lost and not included.

b *n* = without corticosteroids/with corticosteroids.

### 3.3 Biochemical toxicities

Table 3 depicts hematology, electrolytes, and biochemical parameters at different timepoints during and after WBH treatment and the normal pig values (Hannon, Bossone, & Wade, 1990). Concerning hematology, low baseline hemoglobin levels were observed in the animals (5.4 ± 0.8 mmol/L, range 4.1–6.4, normal value 6.2–9.9) (Jackson & Cockcroft, 2002). In animals with 24‐hr postoperative surveillance, a marked decrease up to the lowest value of 2.4 mmol/L in one animal was observed, without the signs of hemolysis (all haptoglobin measurements within normal range). A few animals suffered from substantial blood loss from the invasively placed brain temperature probe. A steady decrease in thrombocyte count during treatment in all animals was observed. This decrease continued in the period post procedure results in a maximal reduction from 186*10^9^/L to 84*10^9^/L in the only animal with available thrombocyte counts up to 24 hr postprocedure (normal value 320–520*10^9^/L [Jackson & Cockcroft, 2002]).

**Table 3:** Hematology, electrolytes, and biochemical parameters at different timepoints during and after WBH treatment (median [interquartile range])

Measurement^b^	Timepoint^a^									
	Normal value pigs ( Jackson & Cockcroft, 2002)	Baseline (t = 0)	Maintenance, middle (t = 7)	Start cooling (t = 11)	Reaching normal temperature (t = 14)	+2 hr (t = 16)	+4 hr (t = 18)	+6 hr (t = 20)	+12 hr (t = 26)	+24 hr (t = 38)
Hb (mmol/L)	6.2–9.9	5.2 [4.7–6.3]	4.9 [4.3–5.6]	5.4 [4.6–5.8]	5.5 [4.3–6.0]	5.2 [3.9–6.2]	5.0 [4.2–5.8]	4.8 [3.8–5.5]	3.8–5.4	3.2–4.3
Leukocytes^c^ (*10^9^/L)	11–22	15.9 [11.0–18.1]	14.3 [11.1–18.1]	13.3 [9.4–17.6]	11.9 [7.3–19.9]	8.2–31.2	7.2–34.2	6.7–34.7	6.7	6.0
Thrombocytes^c^ (*10^9^/L)	320–520	283 [186–305]	210 [161–261]	236 [164–279]	211 [111–284]	135–287	125–257	113–268	114	84
Sodium (mmol/L)	140–150	141 [139–142]	139 [139–141]	139 [138–140]	139 [139–140]	140 [139–141]	142 [139–144]	142 [139–144]	140–150	139–150
Potassium (mmol/L)	4.7–7.1	4.0 [3.9–4.3]	5.3 [5.0–5.8]	5.6 [5.3–6.9]	4.9 [4.6–5.7]	4.8 [4.6–5.3]	4.9 [4.5–5.6]	5.0 [4.4–5.6]	3.8–5.0	3.8–4.6
Ionized calcium (mmol/L)	0.9–1.4	1.30 [1.29–1.34]	1.12 [1.07–1.20]	1.03 [0.97–1.08]	1.09 [1.01–1.18]	1.18 [1.16–1.21]	1.21 [1.17–1.22]	1.19 [1.17–1.24]	—	1.14–1.26
Magnesium (mmol/L)	0.78–1.60	0.72 [0.65–0.76]	0.65 [0.63–0.67]	0.65 [0.58–0.67]	0.68 [0.59–0.72]	0.65 [0.58–0.71]	0.66 [0.59–0.74]	0.69 [0.63–0.75]	0.61–0.72	0.58–0.68
Phosphate (mmol/L)	3.1–5.1	2.63 [2.53–2.73]	2.71 [2.53–2.86]	2.72 [2.65–2.83]	2.62 [2.31–2.82]	2.79 [2.62–3.15]	2.82 [2.71–3.3]	2.96 [2.72–3.44]	2.29–3.52	2.27–2.93
Bicarbonate (mmol/L)	18–27	29.1 [27.6–30.2]	27.5 [25.8–29.6]	28.2 [24.8–29.8]	25.7 [22.9–28.6]	26.0 [23.5–28.7]	25.0 [22.4–29.7]	24.7 [22.8–28.8]	—	28.0–34.4
pH	7.40–7.53 (Hannon et al., 1990)	7.47 [7.44–7.51]	7.41 [7.39–7.47]	7.42 [7.38–7.45]	7.42 [7.36–7.46]	7.43 [7.38–7.49]	7.47 [7.40–7.49]	7.42 [7.36–7.49]	—	7.35–7.52
Lactate (mmol/L)	0.5–1.5	1.5 [1.1–1.7]	1.6 [1.3–2.0]	1.6 [1.3–1.6]	1.6 [1.3–1.9]	1.9 [1.3–2.1]	1.8 [1.3–1.9]	1.5 [1.0–2.8]	—	0.9–1.4
Creatinin (µmol/L)	90–240	106 [99–119]	131 [119–151]	153 [136–168]	154 [132–166]	147 [121–165]	146 [117–176]	139 [118–182]	108–171	100–141
Alkaline phosphatase (U/L)	120–400	101 [91–109]	93 [81–115]	106 [84–122]	105 [84–117]	100 [95–115]	118 [95–135]	145 [113–202]	103–348	102–344
Alanine transaminase (U/L)	31–58	30 [18–34]	19 [15–22]	22 [16–26]	19 [15–26]	23 [16–32]	25 [21–36]	31 [25–39]	25–50	33–63
Aspartate transaminase (U/L)	32–84	30 [25–35]	31 [27–36]	38 [34–52]	50 [42–62]	115 [79–125]	168 [128–208]	209 [166–311]	163–575	335–814
Lactate dehydrogenase (U/L)	380–630	863 [703–1054]	669 [640–729]	711 [655–799]	745 [647–807]	1,112 [929–1191]	1,540 [1020–1734]	1854 [1372–2050]	984–2753	1065–3287
Creatine kinase (U/L)	441–523 (Nonneman et al., 2012)	1,157 [947–1857]	1,009 [750–1705]	1,046 [818–1756]	1,137 [1078–1902]	3,378 [2280–6084]	6,999 [4008–10255]	10,365 [6444–14103]	8428–26540	8500–34210
Glucose (mmol/L)	3.6–5.3	4.5 [3.9–5.2]	7.3 [4.9–7.9]	8.0 [6.3–8.8]	7.6 [6.8–9.1]	6.9 [5.8–10.4]	7.7 [6.3–10.0]	7.9 [7.2–12.0]	6.3–9.9	5.0–7.0

a For animal 1–4 only t = 1–14 and for animal 5, 6, 11, 12 only t = 1–20 available. For t = 26 and t = 38 ranges are depicted (*n* = 4).

b Supplementary Table S1 lists all missing data (not all measurements available at all timepoints for all animals).

c Only available for animal 1–7, from timepoint 16 onwards only ranges (or single values) are given due to limited sample sizes.

**Figure 5:**
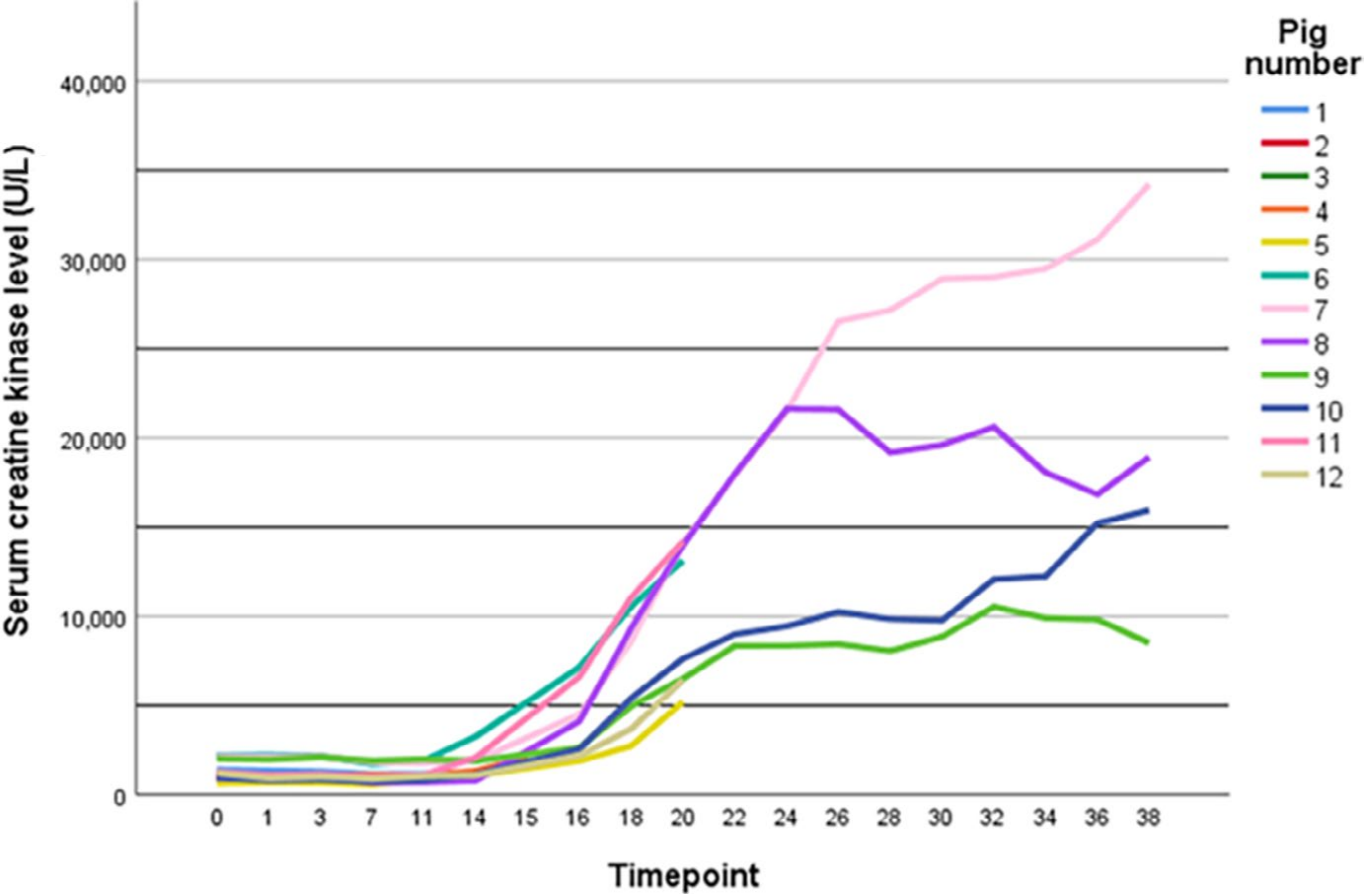
Serum creatine kinase levels during treatment and post procedure Timepoints: t = 0: baseline, t = 1: start of heating, t = 2:15 min after start heating, t = 3: reaching 42°C, t = 4–11: every 15 min in the maintenance phase, t = 12–13:15 and 30 min after cooling started, t = 14: reaching 37°C, t = 15 and higher: hourly in the time post procedure

Mean pH levels remained in the normal range but tended toward alkalosis. Phosphate, calcium, and magnesium levels showed some fluctuations, but returned close to baseline values without intervention. Sodium levels were in normal range and stable over all timepoints. However, severe hyperkalemia was observed (up to 7.4 mmol/L), which led to starting of hemodialysis in that specific animal. In two other animals, dialysis was started to prevent further increase in potassium concentrations. All the three dialyzed animals did not receive corticosteroids. Regarding kidney function, a transient rise in serum creatinine levels from mean 104 ± 21 µmol/L at t = 0 to 143 ± 23 µmol/L during maintenance phase occurred. In the animals with prolonged observation period, at t = 38 (24 hr post procedure) mean creatinine levels had dropped to 121 ± 17 µmol/L (range 100–141 µmol/L). Besides hyperkalemia and transient rise in creatinine concentration, a rapid and ongoing elevation of creatine kinase (CK, up to 34,210 U/L), aspartate transaminase (ASAT, up to 814 U/L), and lactate dehydrogenase (LDH, up to 3,287 U/L) levels in all animals with a prolonged observation period was observed, including the animal that did not receive heating (pig 9), indicating severe rhabdomyolysis (Figure 5).

## 4 DISCUSSION

This study, assessing feasibility of inducing eWBH up to 42°C using the Vithèr WBH device, showed that a blood heating temperature of 45°C delivers an effective and homogenous thermal dose across different organs in a pig model. With this maximal blood heating temperature, brain temperature never exceeded 42.5°C and the highest mean brain temperature during the 2 hr maintenance period was 41.9°C. This is below the critical value of 43°C as assessed by Matsumi et al. This study in vivo assessed thermal damage in monkey brains after local application of hyperthermia at 42–46°C for 60 min. Thermal damage (coagulative necrosis) occurred at temperatures of 44°C or above, but no obvious irreversible damage occurred at lower temperatures (Matsumi et al., 1994). This needs to be interpreted with caution as this study applied hyperthermia for a shorter interval than our study. Besides this, Yarmolenko et al. summarized the effect of different dosages of hyperthermia on several brain parameters (e.g., blood flow, blood–brain barrier, edema, metabolism, direct cell damage) and concluded that the brain tissue sensitivity for thermal damage may be different between species. Pigs seem to have a higher threshold for thermal damage than rabbits and rhesus monkeys. Although in our study brain temperature was invasively monitored and therefore could be robustly assessed over time, no parameters measuring changes in brain function were assessed. This disables drawing conclusions on the safety of this procedure to the brain in this pig model or extrapolation to models with other species (Yarmolenko et al., 2011) In our experiments, vast amounts of intravenous fluids had to be administered to overcome hemodynamic disturbances. Animals receiving corticosteroids tended to perform better regarding hemodynamics and necessity of treatment therefore. Besides hyperkalemia requiring hemodialysis in the three animals (in two to prevent increase in serum potassium; in one to treat hyperkalemia), electrolyte disturbances were mild during eWBH treatment. In the absence of severe renal dysfunction, this hyperkalemia might be explained by severe rhabdomyolysis in all the animals, which was the most worrisome finding in this pig model.

In both animal (Ballard‐Croft et al., 2014; Oglesbee, Diehl, Crawford, Kearns, & Krakowka, 1999; Vertrees et al., 2000) and human (Ash et al., 1997; Cremer et al., 2004; Locker et al., 2011; Zwischenberger et al., 2001) models hemodynamic changes are observed during eWBH treatment. These changes are attributable to a rapid decrease in systemic vascular resistance due to vasodilatation, resulting in a compensatory increase in heart rate and cardiac output (Faithfull et al., 1984; Locker et al., 2011). However, in our study the amount of infused fluids required to maintain adequate arterial blood pressures exceeded the quantities reported in other studies. In one of two animal studies on eWBH performed by Ballard‐Croft et al., a total of 3.070 ± 683 ml (744 ± 205 ml/hr) fluid resuscitation were reported to maintain stable hemodynamics in five sheep during eWBH (2 hr, 42–42.5°C). The second study reported fluid infusion of 100‐500 mL/hr in seven swine undergoing eWBH (2 hr, 42°C) to maintain stable hemodynamics (Ballard‐Croft et al., [Link], 2014). Both studies did not use corticosteroids. A possible explanation of the more pronounced hemodynamic disturbances in our study is the accidental finding of low hemoglobin levels at baseline, of which we do not know the cause. On top of low baseline values of hemoglobin, hemodilution might have aggravated the mismatch between tissue oxygen consumption and blood oxygen transport capacity.

Vasodilatation and hypotension due to loss of systemic vascular resistance, despite high cardiac outputs, is a well‐known complication of cardiopulmonary bypass used in cardiac surgery, called vasoplegia syndrome. The administration of corticosteroids suppresses the systemic inflammatory response associated with this vasoplegia syndrome, leading to better outcomes in a dose‐dependent manner after cardiac surgery (Ho & Tan, 2009; Omar et al., 2015). Therefore, we started to use corticosteroids from animal 8. In our study animals, the administration of 500 mg of methylprednisolone had a favorable effect on hemodynamics, including significantly lower heart rates, higher MAP, and a lower need of fluid infusion. However, due to the small sample size, this has to be interpreted with caution.

Although rhabdomyolysis is a known complication of heat stroke, in the literature of human eWBH, this complication is not described to the same extent as we found in our animal model (Yoshizawa et al., 2016). In humans receiving eWBH, Zwischenberger et al. reported a CK rise up to 1846 ± 1823 U/L 2 hr after the procedure (normal value <170 U/L), Locker et al. described grade 1 (National Cancer Institute Common Toxicity Criteria) CK rise in 33% and grade 2 in 25% of the patients (time interval of occurrence of CK rise not reported) and Zablow et al. also reported mild CK level increases, spontaneously recovering after 2 weeks (only measured after 3 and 14 days) (Locker et al., 2011; Zablow et al., 1997; Zwischenberger et al., 2001). The discordance of the described CK elevations in the literature with our observations might be explained by a pig‐specific stress‐related syndrome. The so‐called porcine stress syndrome describes a situation in which pigs with a mutation in the gene encoding the ryanodine receptor 1 (*RYR1)* respond to stress with malignant hyperthermia and elevated CK levels. Besides stress, inhalational anesthetics (e.g., isoflurane, halothane) are known to trigger porcine stress syndrome in pigs harboring this mutation. However, this mutation is commercially driven largely outbred in most herds to minimize loss of pigs during transport (Nonneman, Brown‐Brandl, Jones, Wiedmann, & Rohrer, 2012; Ritter et al., 2008). It was not tested whether pigs used in this study harbored this specific mutation in the *RYR1* gene, but this is considered unlikely due to the low incidence of this mutation in commercial herds and the presumed absence of genetic relationship between the used animals. However, *RYR1* wild‐type pigs might still be prone to stress syndromes due to other (unknown) genetic deficiencies, especially upon exposure to halothane anesthetics (Allison, Johnson, & Doumit, 2005; Nonneman et al., 2012). In our study, the serum CK level also increased in the animal (pig 9) that underwent extracorporeal circulation without heating, which suggests that this complication may be partially attributable to stress, used anesthetics or extracorporeal circulation and not to hyperthermia treatment itself.

Our study focuses on safety and effects of WBH in otherwise healthy pigs. Due to the absence of tumors in the animals, it is impossible to speculate on the effects of WBH on tumor tissue based on this study. As the proposed beneficial effects of WBH have mostly been extrapolated from studies using locoregional hyperthermia (alone or combined with chemotherapy/radiotherapy), these beneficial effects are still unknown (Hildebrandt, Wust, & Ahlers, 2002). Confirmation of the assumption that WBH exerts the same beneficial antitumor effects as locoregional hyperthermia is warranted. Confirmation of these beneficial antitumor effects of WBH (e.g., clinical responses, improvement of overall survival) could provide a rationale for application of WBH in patients with metastasized malignancies lacking other treatment options.

A strength of our experiment, as described earlier, is the adaptive design as part of our learning curve. By forming the hypotheses based on interim analyses, we succeeded to mitigate some of the observed complications with prudent use of animals. Nevertheless, serious complications as rhabdomyolysis and probably as a consequence hyperkaliemia, were troublesome, necessitating restraint extrapolation to human experiments. Studies using comparable WBH techniques in humans in, respectively, six HIV patients, five patients with non–small cell lung cancer, and six patients with metastatic sarcoma, reaching a body temperature of up to 42°C–42.5°C for 2 hr, did not encounter these features at the same magnitude (Locker et al., 2011; Zablow et al., 1997; Zwischenberger et al., 2001).

In conclusion, we present a feasible method of inducing eWBH, using the Vithèr Hyperthermia B.V. WBH device, in pigs with a homogenous thermal distribution across different locations in the body, including the brain. Severe hemodynamic disturbances were observed, which might be solved with the administration of corticosteroids. Another treatment‐limiting toxicity was rhabdomyolysis, possibly due to a pig‐specific stress reaction. Therefore, a translation of these results to patients with metastasized cancer must be done with great caution and with special attention for hemodynamics as well as rhabdomyolysis. This can best be performed in a phase I study with patients who have no other treatment options left, and who have an adequate general condition.

## CONFLICT OF INTEREST

GL, TF, MAJ, JGvdH, CvH, and GJS: Nothing to declare; MM is initiator of the eWBH project and co‐owner of the Vithèr WBH device.
