# Supplementary material for: Thermal distribution, physiological effects and toxicities of extracorporeally induced whole‐body hyperthermia in a pig model
Source: Physiol Rep. 2020 Feb 25;8(4):e14366. doi: 10.14814/phy2.14366 (PMC7058172; doi:10.14814/phy2.14366)
Supplement: Supplementary file 1 [file PHY2-8-e14366-s001.pdf]

Appendix/supplementary materials

Supplementary table 1. Missing data which are not included in the analysis and tables/figures.

**Pig number**	**Timepoint number**	**Assessment**
		**Thermal data**
1-7	All	Airway
3	All	Left auditory canal
		**Vital signs**
1	2,14	HR, MAP
2	11,15	HR, MAP
3	2	HR, MAP
8	6,33	MAP
8	33,34	HR
9	10	HR
11	2	HR,MAP
		**Biochemical**
1	All	lactate
1	11	All
2	0	Lactate
2	7	AF, ASAT, ALAT, LD, CK, haptoglobin
5	0	Ionized calcium, bicarbonate, pH, lactate
5	11	Creatinine
7	18	Bicarbonate, pH, lactate, ionized calcium
7	20	Bicarbonate, pH, lactate, ionized calcium
7-10	26	Bicarbonate, pH, lactate, ionized calcium
8,10-12	all	Leukocytes/thrombocytes
8	16	Hb
9	all	Thrombocytes
9	0,14,16,18,20,26,38	Leukocytes
10	20	Bicarbonate, pH, lactate, ionized calcium
11	0	LD
